# Establishment of a regression model of bone metabolism markers for the diagnosis of bone metastases in lung cancer

**DOI:** 10.1186/s12957-021-02141-5

**Published:** 2021-01-24

**Authors:** Zhongliang Zhu, Guangyu Yang, Zhenzhen Pang, Jiawei Liang, Weizhong Wang, Yonglie Zhou

**Affiliations:** Department of Clinical Laboratory, Zhejiang Provincial People’s Hospital, People’s Hospital of Hangzhou Medical College, Hangzhou, 310014 Zhejiang China

**Keywords:** Bone metabolism markers, Lung cancer, Bone metastases

## Abstract

**Background:**

The aim of this study was to establish a regression equation model of serum bone metabolism markers. We analyzed the diagnostic value of bone metastases in lung cancer and provided laboratory evidence for the early clinical treatment of bone metastases in lung cancer.

**Methods:**

A total of 339 patients with non-metastatic lung cancer, patients with lung cancer with bone metastasis, and patients with benign lung disease who were treated in our hospital from July 2012 to October 2015 were included. A total of 103 patients with lung cancer in the non-metastatic group, 128 patients with lung cancer combined with bone metastasis group, and 108 patients with benign lung diseases who had nontumor and nonbone metabolism-related diseases were selected as the control group. Detection and analysis of type I collagen carboxyl terminal peptide β-special sequence (β-CTX), total type I procollagen amino terminal propeptide (TPINP), N-terminal-mid fragment of osteocalcin (N-MID), parathyroid hormone (PTH), vitamin D (VitD3), alkaline phosphatase (ALP), calcium (CA), phosphorus (P), cytokeratin 19 fragment (F211), and other indicators were performed. Four multiple regression models were established to determine the best diagnostic model for lung cancer with bone metastasis.

**Results:**

Analysis of single indicators of bone metabolism markers in lung cancer was performed, among which F211, β-CTX, TPINP, and ALP were significantly different (*P* < 0.05). The ROC curve of each indicator was less than 0.712. Based on the multiple regression models, the fourth model was the best and was much better than a single indicator with an AUC of 0.856, a sensitivity of 70.0%, a specificity of 91.0%, a positive predictive value of 82.5%, and a negative predictive value of 72.0%.

**Conclusion:**

Multiple regression models of bone metabolism markers were established. These models can be used to evaluate the progression of lung cancer and provide a basis for the early treatment of bone metastases.

## Background

Lung cancer is one of the most common malignant tumors, and its morbidity and mortality remain high. The incidence of lung cancer has leapt to the top in the world [1, 2]. Clinically, recommended guidelines for the diagnosis of bone metastases in lung cancer show that identification of bone metastases in lung cancer primarily relies on imaging findings. According to relevant statistics, approximately 70% of lung cancer patients are often in the middle or advanced stages when bone metastases occur, at which point they have lost the opportunity to undergo radical surgery for lung cancer. The average 5-year expected survival rate is not ideal. At present, there are few clinically effective prediction methods for bone metastases in lung cancer [3–5]. Studies have shown that different types of molecules, host cells, and the extracellular microenvironment participate in cancer cell interactions during bone metastasis in lung cancer, including osteoclast-mediated bone resorption and osteoblast-mediated bone formation. Sometimes they also interact with each other [6–8]. Therefore, we speculate that biochemical bone metastases are likely to occur before imaging bone metastases. To further improve the level of prediction, diagnosis, and disease monitoring of bone metastases in lung cancer [9], this study used existing clinical laboratories to detect biochemical markers of bone metabolism, conduct joint analysis, and establish logistic regression equations for multiple combined models. We sought to determine the early diagnostic value of bone metastases in lung cancer.

## Methods

### Patients

A total of 339 patients with non-metastatic lung cancer, patients with lung cancer with bone metastases, and patients in the benign disease control group were enrolled from July 2012 to October 2015. One hundred three cases of lung cancer without bone metastasis (hereinafter referred to as the non-metastatic group), including 64 males and 39 females, with an average age of 64 ± 12 years were included. One hundred twenty-eight cases of lung cancer combined with bone metastasis (hereinafter referred to as the metastasis group), including 79 males and 49 females, with an average age of 64 ± 11 years were included. One hundred eight cases with nontumor and nonbone metabolism-related diseases were selected as controls (hereinafter referred to as the control group), including 67 males and 41 females with an average age of 65 ± 12 years. The present study was approved by the Ethics Committee of the hospital, and as a retrospective study, the requirement for informed patient consent was waived (Table 1).

**Table 1:** Demographics of the three groups of patients

Characteristics	Non-metastatic (***n*** = 103)	Metastatic (***n*** = 128)	Control (***n*** = 108)
Age (years)	64 ± 12	64 ± 11	65 ± 12
Female	39	49	41
Male	64	79	67
F/M	0.61:1	0.62:1	0.61:1

### Diagnostic and exclusion criteria

The diagnostic criteria for lung cancer with bone metastasis are the “Expert Consensus on the Diagnosis and Treatment of Bone Metastasis in Lung Cancer (2019 version).” It should meet one of the following two conditions [10]: ① a clinical or pathological diagnosis of lung cancer and bone biopsy showing lung cancer metastasis; ② the pathological diagnosis of lung cancer is clear, and the imaging examination has typical bone metastases. Imaging examinations included ECT, MRI, PET-CT, CT, and X-rays. Exclusion criteria for lung cancer bone metastasis should meet one of the following two conditions: ① clinically diagnosed as other malignant tumors; ② clinically diagnosed bone metabolic diseases include osteoporosis, endocrine bone disease, renal bone disease, deformable osteitis, and hereditary bone disease. Basic case information, such as age, sex, time of onset, laboratory indicators, and imaging reports, was collected according to the selected and excluded indicators.

### Main experimental reagents and equipment

The β-CTX determination kit, TPINP determination kit, N-MID determination kit, PTH determination kit, VitD3 determination kit, and F211 determination kit were provided by Roche Diagnostics, Switzerland; the ALP determination kit, CA determination kit, and P determination kit were provided by Beckman-Coulter, USA. The E170 automatic immunoluminescence instrument and automatic sample preprocessing pipeline system were provided by Roche Diagnostics, Switzerland. The Olympus AU5400 automatic biochemical analyzer was provided by Beckman-Coulter, and an MDF-U281 low-temperature refrigerator was provided by Japan Sanyo Corporation.

### Detection method

All patients required fasting for more than 10 h. The following morning, 5 ml of fasting elbow venous blood was drawn and loaded into a BD yellow tube. After coagulation, the samples were centrifuged at 2465 (×*g*) for 10 min, and then the serum was detected according to the requirements of the reagent instructions. We analyzed bone metabolism markers, found indicators with significant differences between groups, and excluded indicators without significant differences. We calculated the sensitivity, characteristics, and critical points and evaluated the diagnosis of each indicator performance. Multifactor regression analysis was used to establish multiple logistic regression models of combined models to evaluate their diagnostic performance for lung cancer bone metastases.

### Statistical analysis

SPSS 16.0 statistical software was used for statistical analysis. P and CA are expressed as the means ± standard error of the mean. Statistical analysis was performed using Student’s *t* test. β-CTX, TPINP, N-MID, PTH, VitD3, ALP, and F211 are expressed as the non-normal distribution errors. Statistical analysis was performed using nonparametric tests. Logistic regression analysis was used to establish a diagnostic model (inclusion criteria: *P* < 0.05, exclusion criteria: *P* ≥ 0.05). *P* < 0.05 was considered to indicate a statistically significant difference.

## Results

### Bone metabolism marker characteristics

**Fig. 1:**
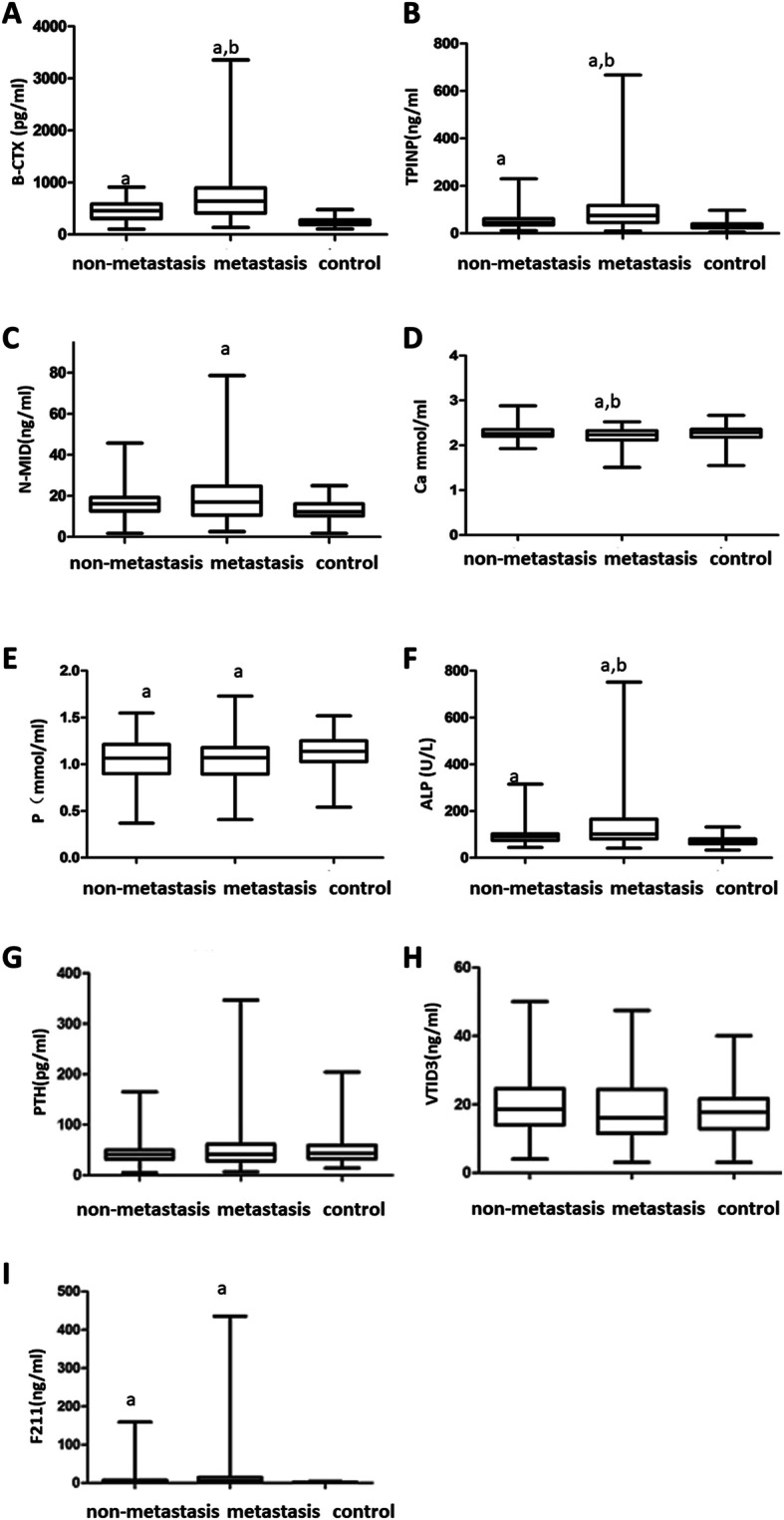
The units for the indicator are as follows: P (mmol/L), CA (mmol/L), PTH (pg/ml), ALP (U/L), F211 (ng/ml), β-CTX (pg/ml), VITD3 (ng/ml), TPINP (ng/ml), and N-MID (ng/ml). (a) Metastasis group compared to the control group (*P* < 0.05). (b) Metastasis group compared to the non-metastatic group (*P* < 0.05)

We explored the clinical relevance of various indexes in the lung cancer bone metastasis, lung cancer non-metastasis, and control groups (Fig. 1). The levels of P, ALP, β-CTX, TPINP, and F211 in the lung cancer non-metastatic group were significantly different from those in the control group (*P* < 0.05). The levels of P, CA, ALP, β-CTX, TPINP, N-MID, and F211 in the lung cancer bone metastasis group were significantly different from those in the control group (*P* < 0.05). Furthermore, the levels of CA, ALP, β-CTX, and TPINP in the lung cancer bone metastasis group were significantly different from those in the non-metastatic lung cancer group (*P* < 0.05).

### Single marker analysis

We first examined the diagnostic performance of single markers in bone metastases of lung cancer. The performance of various indicators for diagnosis is shown in Table 2. The selected indicators were F211, β-CTX, TPINP, and ALP. The areas under the curve (AUCs) were 0.588, 0.687, 0.712, and 0.635, respectively. Among them, the AUC of TPINP was the largest at 0.712. The sensitivity of TPINP for the diagnosis of bone metastasis was 60.9%, and the specificity was 78.4%.

**Table 2:** Diagnostic performance of various indicators

Indicator	Cut-off	AUC	Sensitivity	Specificity	PPV	NPV
F211	7.400	0.588	0.502	0.765	0.660	0.667
β-CTX	601.000	0.687	0.555	0.794	0.649	0.451
TPINP	64.200	0.712	0.609	0.784	0.574	0.451
ALP	122.500	0.635	0.523	0.882	0.711	0.600
N-MID	19.350	0.543	0.409	0.767	0.186	0.781
PTH	65.300	0.529	0.236	0.913	0.088	0.922
Ca	2.385	0.406	0.142	0.864	0.324	0.727
P	0.945	0.499	0.717	0.032	0.000	1.000
VITD3	30.550	0.422	0.165	0.903	0.100	0.875

### Multiple regression model analysis

For multivariate regression analysis, β-CTX, TPINP, CA, and ALP were selected (Table 3), and logistic regression models were established: model 1: β-CTX+TPINP; model 2: β-CTX+TPINP+CA; model 3: β-CTX× TPINP; and model 4: β-CTX×TPINP + ALP + CA.

**Table 3:** Diagnostic performance of the models

Models	Cut-off	AUC	Sensitivity	Specificity	PPV	NPV
p1	17.300	0.836	0.781	0.740	0.617	0.641
P2	7.350	0.813	0.788	0.773	0.740	0.680
p3	26645.460	0.829	0.785	0.825	0.654	0.718
p4	4.480	0.856	0.700	0.910	0.825	0.720

Logistic model regression equations are as follows:

p1 = − 1.764 + 2 × 10^−2^ (β-CTX) + 1.6 × 10^−2^ (TPINP)

p2 = 4.645 + 2 × 10^−2^(β-CTX) − 2.58(CA) + 1.6 × 10^−2^ (TPINP)

p3 = β-CTX × TPINP

p4 = 5.011 + 2.6 × 10^−5^(β-CTX × TPINP) + 4.10^−2^ × (ALP) − 2.779(CA)

## Discussion

Lung cancer remains the leading cause of cancer-related mortality worldwide. Bone metastases from lung cancer cause great pain to patients. X-ray is the most convenient and economical routine modality for bone examination [11, 12]. However, it is not sensitive to small bone metastases less than 1 cm. Single-photon emission computed tomography (SPECT) also shows a dense phenomenon for bone trauma, inflammation, degeneration, and other lesions, which is easily misdiagnosed as cancerous bone metastasis, leading to false positives [13]. Magnetic resonance imaging (MRI) is performed for metastases that cannot be clearly identified. However, with in-depth study of the mechanism of bone metastasis, we found that we can monitor changes in bone metabolism biochemical markers to determine whether lung cancer is associated with bone metastases [14].

After reasonable and scientific screening of bone metabolism markers and monitoring their levels [15], it was found that there was a correlation with bone metastases in lung cancer [16, 17]. Studies have shown that lung cancer stem cells transfer lung cancer cells to the bone in three steps: the first step is to escape from the primary tumor, the second step is to circulate, and the third step is to settle in the bone. Lung cancer bone metastasis is a complex cellular biological transformation in which there are interactions with host cells and the extracellular microenvironment by the cancer cells [18]. β-CTX is a marker of bone resorption. It can be detected when released into the blood after osteolysis and is used to detect bone metastases. TPINP is a marker of bone formation. Levels of TPINP in the blood are positively correlated with the levels of collagen synthesized by osteoblasts, which reflect the activity of osteoblasts. TPINP is not easily degraded in the blood and is less affected by hormones. It is used as a follow-up indicator for monitoring bone metastases.

Our study observed that the bone metabolism markers β-CTX, TPINP, N-MID, P, CA, and ALP were significantly different between the lung cancer with bone metastasis group and the non-metastatic group. Markers are involved in the process of bone metastasis. Among them, the sensitivity of β-CTX was only 60.2%, the specificity was 79.4%, the positive predictive value was 56.3%, and the negative predictive value was 45.1%. The sensitivity of TPINP was 60.9%, the specificity was 78.4%, the positive predictive value was 57.4%, and the negative predictive value was 45.1%. These findings illustrate that the performance of simultaneous positive prediction and negative prediction using a single indicator is poor. In this paper, multivariate logistic regression analysis was performed for each individual bone metabolism marker. Four different combination models were established. The sensitivity of combination 4 was 70.0%, the specificity was 91.0%, and the positive predictive value was 82.5%, while the negative predictive value was 71.8%. It follows that the diagnostic performance of the combined fourth model is the best. The combined fourth model reflects changes in the activity of osteoblasts and osteoclasts [19–21]. These indicators can be easily identified by testing the blood. Clinically, this fourth model can be used to evaluate the progression of lung cancer and provide a basis for the early treatment of bone metastases.

## Conclusion

Establishing multiple regression models of bone metabolism markers represents a method to evaluate the progression of lung cancer and provides a basis for the early treatment of bone metastases.

## Data Availability

The analyzed datasets generated during the study are possibly available from the corresponding author on reasonable request.

## Abbreviations

TPINP: Total type I procollagen amino terminal propeptide
β-CTX: Type I collagen carboxyl terminal peptide β-special sequence
N-MID: N-Terminal-mid fragment of osteocalcin
PTH: Parathyroid hormone
ALP: Alkaline phosphatase
CA: Calcium
P: Phosphorus
F211: Cytokeratin 19 fragment
MRI: Magnetic resonance imaging
SPECT: Single-photon emission computed tomography

## References

[1] Salomaa ER, Walta M (2015). The prognosis of lung cancer continues to be poor-treatment outcome within the hospital district of Southwest Finland in 2004 to 2011. Duodecim..

[2] GLOBOCAN. Estimated cancer incidence, mortality and prevalence worldwide in 2012.IARC. 2014.

[3] Hess KR, Varadhachary GR, Taylor SH (2006). Metastatic patterns in adenocarcinoma. Cancer..

[4] Paget S (1989). The distribution of secondary growths in cancer of the breast. Cancer Metastasis Rev..

[5] Uy HL, Mundy GR, Boyce BF (1997). Rood man GD and Guise TA: Tumor necrosis factor enhances parathyroid hormone-related protein-induced hypercalcemia and bone resorption without inhibiting bone formation in vivo. Cancer Res..

[6] Zhang Y, Biswas S (2015). An improved version of logistic Bayesian LASSO for detecting rare haplotype environment interactions with application to lung cancer. Cancer Inform..

[7] Bogenrieder T, Herlyn M (2003). Axis of evil: Molecular mechanisms of cancer metastasis. Oncogene..

[8] Cai Z, Chen Q, Chen J (2009). Monocyte chemotactic protein 1 promotes lung cancer-induced bone resorptive lesions in vivo. Neoplasia..

[9] Karapanagiotou EM, Terpos E, Dilana KD (2010). Serum bone turnover markers may be involved in the metastatic potential of lung cancer patients. Med Oncol..

[10] Youth Specialists Committee of Lung Cancer (2019). Beijing Medical Award Foundation, Chinese Lung Cancer Union. Zhongguo Fei Ai Za Zhi..

[11] Homann G, Mustafa DF (2015). Improved detection of bone metastases from lung cancer in the thoracic cage using 5-and 1-mm axial images versus a new CT software generating rib unfolding images: comparison with standard (1)(8)F-FDG-PET/CT. Acad Radiol..

[12] Inal, A, M. A. Kaplan, et al. Is there any significance of lung cancer histology to compare the diagnostic accuracies of (18) F-FDG-PET/CT and (99 m) Tc-MDP BS for the detection of bone metastases in advanced NSCLC? ContempOnco. 2014; l18: 106-110. 10.5114/wo.2014.42725 PMC4068819 24966793

[13] Rachel KL, Borondy A, Tremblay A (2020). Lung cancer screening effective for reducing cancer deaths. American family physician.

[14] Mulshine JL, Avila RS, Conley E (2020). The International Association for the Study of Lung Cancer Early Lung Imaging Confederation. JCO Clin Cancer Inform..

[15] Meng C, Tang C, Liang J (2018). Progress of biomarkers in diagnosis of bone metastases of lung cancer. Chinese journal of lung cancer..

[16] Niu Y, Lin Y, Pang H (2019). Risk factors for bone metastasis in patients with primary lung cancer: a systematic review. Medicine..

[17] Confavreux CB, Pialat JB, Bellière A (2019). Bone metastases from lung cancer: a paradigm for multidisciplinary onco-rheumatology management. Joint Bone Spine..

[18] Hiraga T (2019). Bone metastasis: interaction between cancer cells and bone microenvironment. Oral Biosci..

[19] Lorenza Landi, Federica D'Incà, Alain Gelibter, et al. Bone metastases and immunotherapy in patients with advanced non-small-cell lung cancer. Journal for immunotherapy of cancer. 2019; 1:316. 10.1186/s40425-019-0793-8 PMC6868703 31752994

[20] Zhang JY, Ge P, Zhang PY (2019). Role of neutrophil to lymphocyte ratio or platelet to lymphocyte ratio in prediction of bone metastasis of prostate cancer. Clin Lab..

[21] Dewulf J, Vangestel C, Verhoeven Y (2019). Bone metastases in the era of targeted treatments: insights from molecular biology. Nucl Med Mol Imaging..

